# Comparative Corneal Histomorphometry Between Birds of Different Species

**DOI:** 10.3390/biology14060603

**Published:** 2025-05-25

**Authors:** Rafaela A. R. Tozetti, Matheus V. L. Moreira, Rosélia L. S. Araújo, Liria Q. L. Hirano, Bret A. Moore, Paula D. Galera

**Affiliations:** 1 Post-Graduate Program in Animal Sciences, Faculty of Agronomy and Veterinary Medicine, University of Brasília, Brasília 70910-900, Brazil; rafaelartozetti@gmail.com (R.A.R.T.); roselialaraujo@hotmail.com (R.L.S.A.); 2 MVL Patologia Veterinária, Belo Horizonte 31330-572, Brazil; mvlpatologiaveterinaria@gmail.com; 3 Department of Wild Animals, Faculty of Agronomy and Veterinary Medicine, University of Brasília, Brasília 70910-900, Brazil; liriahirano@unb.br; 4 Department of Small Animal Clinical Sciences, College of Veterinary Medicine, University of Florida, Gainesville, FL 32611, USA; bretskimoore@gmail.com

**Keywords:** avian, eye, histology, morphometry, ophthalmology, vision

## Abstract

To describe the corneal histomorphometry of 8 birds from different species, this study analyzed 11 eyes from individuals that had recently died due to various causes. Only healthy eyes were included, representing the following species: *Asio stygius*, *Crotophaga ani*, *Pitangus sulphuratus*, *Turdus rufiventris*, *Ramphastos toco*, *Rhea americana*, *Ara macao*, and *Nyctibius griseus*. The eyes were enucleated within 30 min after death and fixed in 10% formaldehyde. The corneas were analyzed by light microscopy to measure and describe their layers. While the general structure and composition were consistent with those of other vertebrates, significant differences were found among species in the thickness of the corneal layers. These differences were observed between the central and peripheral regions of the same cornea, as well as between species. The central region was predominantly thinner than the periphery. Epithelial thickness varied from 2% to 16% of the total corneal thickness. The number of epithelial layers also varied between species, ranging from three to six. Bowman’s layer was present in all birds studied, and the stroma was the thickest layer in every sample. These findings contribute to a better understanding of the corneal morphology in diverse bird species and may support future ophthalmic or comparative anatomical research.

## Simple Summary

The cornea is a crucial part of the eye, responsible for protecting its interior and assisting in focusing and refracting images. To better understand the visual and structural differences between bird species, this study aimed to describe the corneal layers and their measurements in several birds. Using a simple and effective method, light microscopy, it was possible to observe and describe the microscopic structure of the birds’ corneas. The results revealed that while the overall corneal composition is consistent across all birds, there are specific differences in the metrics for each species. All birds exhibited variations in the total thickness of the cornea and its layers between the central and peripheral regions, with the central region being thinner than the peripheral region in most samples. In the Stygian owl, the variation between these regions is greater compared to the other species. Among bird species, there were also significant variations in epithelial thickness and the number of epithelial layers. This improved understanding of eye structures helps explain how birds visually interact with their environment and enhances the ability to interpret pathological changes in bird corneas.

## 1. Introduction

The visual system of birds varies as much as the broad variety of species within this taxon, where the morphology and physiology of vision meet the needs for success in the specific ecological niche they occupy. Corneal morphometry has been studied in different vertebrate species besides birds, including dogs, cats, horses, camels, and non-human primates [1,2,3,4,5,6]. The vertebrate ocular surface includes the cornea, a transparent tissue that aids in image formation, refractive power, ocular protection, and mechanical support [7,8,9,10]. Total corneal thickness is a primary focus of research, and this measurement can be obtained using different microscopy techniques. Scientific interest in this area extends beyond veterinary medicine to include human medicine and biological sciences [11,12,13,14]. Despite similarities across vertebrates, morphological differences are readily observable across species, including those sharing the same habitat [5,15].

In the central region of Brazil, there are several bird species that share the same ecosystem but have different habits and play distinct roles, leading to variations in corneal structures [13,16,17,18]. Some avian species from this region include Scarlet Macaws (*Ara macao*) and Toco Toucans (*Ramphastos toco*), which mainly inhabit forests and feed on fruits, seeds, and insects [19,20,21]. The Rufous-bellied Thrush (*Turdus rufiventris*) and the Great Kiskadee (*Pitangus sulphuratus*) are two examples of passerines; while belonging to different families, both have an excellent adaptation to urban life [22,23,24,25,26]. The Greater Rheas (*Rhea americana*) are omnivorous birds that do not have the ability to fly, and they differ from most other birds in that they have proportionally smaller eyes when compared to their skulls [27,28]. The Smooth-billed Ani (*Crotophaga ani*) is a cuculiform, a diurnal bird that feeds on insects and arthropods, and it can catch small fish, usually grabbing prey in mid-flight [29,30,31]. Stygian Owls (*Asio stygius*) and Common Potoos (*Nyctibius griseus*) are nocturnal birds with large eyes, which provide high visual sensitivity in dim environments [32,33,34].

The corneas of birds are composed of five histological layers: epithelium, Bowman’s layer, stroma, Descemet’s membrane, and endothelium. However, in some birds, the Bowman’s layer has not been identified [1,3,10,35]. Although the gross arrangement and structure of the cornea does not differ among most birds, a distinction can be seen in the thickness and components of their corneal layers [36,37,38]. Due to the plurality of avian species, seeking knowledge of interspecific differences could help to better understand their visual demands, behaviors, and ecology. This study aimed to describe the histomorphometry of the healthy cornea of eight phylogenetically diverse avian species, chosen according to availability upon presentation, from the Midwest region of Brazil: *Asio stygius*, *Crotophaga ani*, *Pitangus sulphuratus*, *Turdus rufiventris*, *Ramphastos toco*, *Rhea americana*, *Ara macao*, and *Nyctibius griseus*.

## 2. Materials and Methods

### 2.1. Animals

Eight adult birds of different species were included in this study. Only healthy eyes were considered, totaling 11 eyes. Two eyes of a Stygian Owl (*Asio stygius*), two eyes of a Smooth-Billed Ani (*Crotophaga ani*), one eye of a Great Kiskadee (*Pitangus sulphuratus*), one eye of a Toco Toucan (*Ramphastos toco*), one eye of a Rufous-bellied Thrush (*Turdus rufiventris*), one eye of a Greater Rhea (*Rhea americana*), one eye of a Scarlet Macaw (*Ara macao*) and finally, two eyes of a Common Potoo (*Nyctibius griseus*) (order, family and popular names described in Table 1). The birds included in this study were housed at the Department of Wild Animals of the Faculty of Agronomy and Veterinary Medicine at the University of Brasília after being rescued by the Federal District’s Wild Animal Screening Center (*Centro de Triagem de Animais Silvestres do Distrito Federal—CETAS*). The collection of biological material was authorized by the Biodiversity Authorization and Information System (*Sistema de Autorização e Informação em Biodiversidade—SISBIO*), under protocol number SISBIO n.79141-2. All animals received veterinary evaluation by a multidisciplinary team, including an ophthalmologic examination performed by the ophthalmology service of the University of Brasília Veterinary Hospital. The ophthalmic assessment included anterior segment and adnexa evaluation using a slit-lamp biomicroscope Kowa SL-15 (Kowa Company, Ltd., Nagoya, Japan), posterior segment evaluation with direct ophthalmoscopy PanOptic (Welch Allyn, Inc., Skaneateles Falls, NY, USA), and intraocular pressure measurement using rebound tonometry TonoVet (Icare Finland Oy, Vantaa, Finland). Only birds that died naturally due to causes unrelated to ocular conditions were included. Eyes were collected only from individuals that showed no clinical signs of ophthalmic disease, such as uveitis, hyphema, corneal ulcers, globe perforation, or posterior segment abnormalities.

### 2.2. Sample Collection and Processing

The eyes were removed no later than 30 min after the bird’s death, eliminating the possibility of degeneration or freezing artifacts. Although this method limited the number of bird specimens, as it was necessary to collect them as quickly as possible after the natural death of the animals, it limited the amount of potential artifacts. A transconjunctival technique was utilized to remove the eyes, which consisted of a 360° perilimbal incision, dissection of the ocular attachments to isolate the globe, and transection of the extraocular muscles and optic nerve. The eyes were placed in a 10% formaldehyde solution and sent to the Veterinary Pathology Laboratory (MVL Patologia Veterinária, Belo Horizonte, Brazil), where the samples were processed and analyzed by light microscopy.

### 2.3. Tissue Preparation

Eyes were processed according to conventional histological techniques [39]. They were dehydrated using a graded series of alcohol (70–100%), followed by clarification using xylene before being embedded in paraffin. Subsequently, the blocks containing the samples were subjected to serial cuts with a thickness of 4 μm, which were placed on slides and stained with hematoxylin and eosin (HE).

### 2.4. Histological Analysis and Description

Tissue samples were evaluated using an Opticam O500R (Opticam, São Paulo, Brazil) light microscope and OPTHD software (Opticam Microscopia, version: x64, 4.7.15283.20190804). For birds that had both eyes evaluated, the average value of both corneas was calculated. The following structures were identified and measured in the central region and periphery of the cornea: Central Cornea Full Thickness (CCFT), Peripheral Corneal Full Thickness (PCFT), Central Epithelium (CEp), Peripheral Epithelium (PEp), Central Bowman’s Layer (CBL), Peripheral Bowman’s Layer (PBL), Central Stroma (CS), Peripheral Stroma (PS), Central Descemet’s Layer (CDL), and Peripheral Descemet’s Layer (PDL). Values are given in micrometers (µm). Endothelial thickness was not measured, as it was lost during histological processing.

## 3. Results

**Figure 1:**
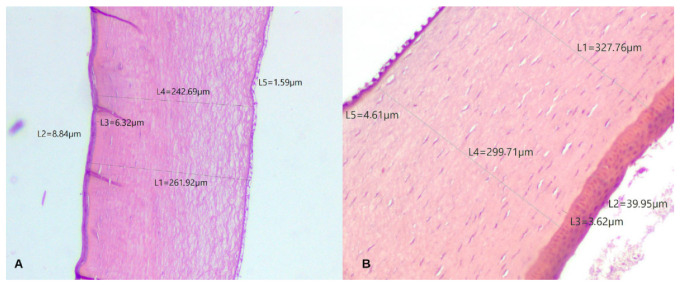
Photomicrographs showing measurements of total corneal thickness (L1) and its individual layers: Epithelium (L2), Bowman’s layer (L3), stroma (L4), and Descemet’s membrane (L5). Central corneal region of (**A**) *Asio stygius* and (**B**) *Rhea americana*. Magnification 100×, stained with hematoxylin and eosin.

The evaluated corneas revealed an avascular tissue composed of four layers as previously described in other species, from anterior to posterior: the epithelium, Bowman’s layer, stroma, and Descemet’s membrane. The internal endothelium was absent due to histological processing. Total corneal thickness varied between bird species and between the corneal regions. The central region of the total cornea was thinner than the peripheral region in *Asio stygius*, *Crotophaga ani*, *Turdus rufiventris*, *Ramphastos toco*, *Rhea americana*, and *Nyctibius griseus*; it was thicker in *Pitangus sulphuratus* and *Ara macao*. Table 2 summarizes the measurements of the corneal layers, and Figure 1 illustrates the histomorphometry of the corneas from both the Greater Rhea and the Stygian.

**Figure 2:**
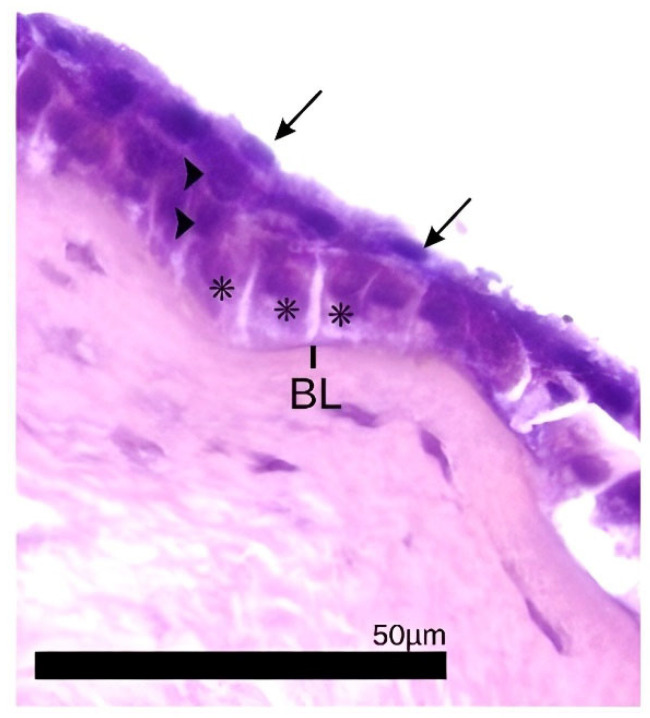
Photomicrograph of a *Turdus rufiventris* cornea. Columnar-shaped basal epithelial squamous cells (asterisk), polyhedral cells (arrowhead), wing cells (arrows), and Bowman’s layer (BL) are evident. Magnification 400×, stained with hematoxylin and eosin.

The epithelium consisted of nonkeratinizing stratified squamous cells. The number of epithelial layers is species-specific and corneal-region-specific, and in this study, it ranged from three to six cell layers with one layer of posterior basal cells, one to three layers of middle polyhedral squamous cells, and one to three layers of anterior flattened squamous cells (Figure 2). The number of layers and the thickness of the epithelium varied between the central and peripheral regions of the cornea. Table 3 shows the number of epithelial layers in each corneal region of the studied species.

Bowman’s layer was observed in all eight birds studied. It also showed variation in its thickness in the center compared to the periphery, being thinner in the central cornea in *Turdus rufiventris*, *Ramphastos toco*, *Rhea americana*, *Ara macao*, *Pitangus sulphuratu*, and *Nyctibius griseus* (Table 2). The stroma was the thickest portion of the cornea in the birds studied. Except for *Pitangus sulphuratu* and *Ara macao*, all birds had a central stroma thinner than the periphery (Table 2). Attached to the innermost part of the stroma, the Descemet’s membrane thickness varied slightly between species and between central and peripheral regions of the cornea (Table 2).

## 4. Discussion

**Figure 3:**
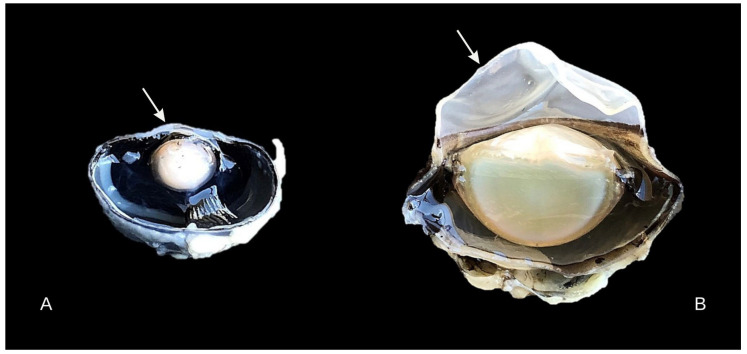
Gross photography of hemisected eyes demonstrating the variation of the corneal curvature (arrow) between *Crotophaga ani* (**A**) with a low degree of curvature, and of *Nyctibius griseus* (**B**), with high convexity.

Whole corneal morphometry has been relatively understudied, with the total thickness of the cornea being the most common measurement among researchers and clinicians. Through different microscopy models, corneal thicknesses can be obtained, and the scientific purposes span across not only veterinary medicine, but also human medicine and the biological sciences [11,12,13,14]. Table 4 summarizes the results of previous studies as well as the findings in this study, and it shows that corneal morphology varies widely among birds. There is likely a correlation between habitat and morphological differences in the cornea [10,32,40]. The total thickness of the cornea can also vary significantly between animals of the same species, in relation to age, breed, and regions of the cornea [16,41]. Slight variations can even be seen between left and right eyes and between males and females [18]; however, in the present study, the sex of the birds was not considered as an evaluation criterion. The curvature of the cornea is also correlated with the thickness (Figure 3); birds with a flatter cornea have little difference between the center and the periphery [36]. On the other hand, in birds with a large corneal curvature, such as the Golden Eagle (*Aquila chrysaetos*) [42], and the Stygian Owl (*Asio stygius*) and Common Potoo (*Nyctibius griseus*) in this study, the periphery is substantially thicker than the center [43,44].

**Figure 4:**
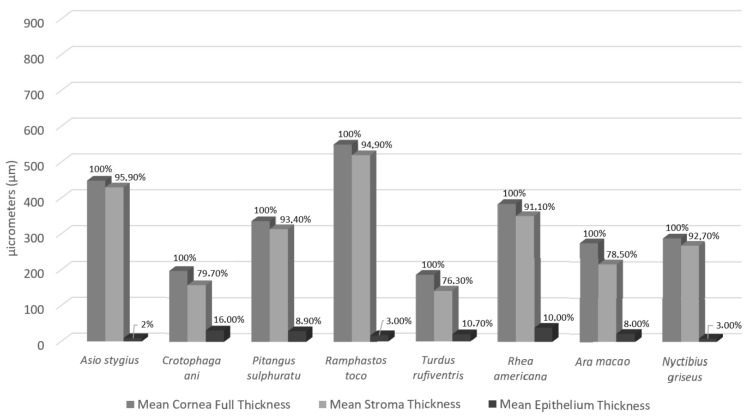
Proportional representation of corneal epithelium and stroma thickness as a percentage of the total corneal thickness (100%) in each of the studied avians.

Not only does the total thickness of the cornea vary, but the individual corneal layers vary in thickness as well. The epithelium is the corneal anterior surface, and it is composed of layers of stratified squamous and non-keratinized cells. The basal cell layer has a cuboidal or columnar shape, covered by multiple layers of cells that become wider and flatter as they move away from the base, which are polyhedral. The most superficial squamous cells are almost completely flattened; they are called umbrella or wing cells, as their extent overlaps the apices of more than one cell. This epithelial pattern is found in previously studied mammals and birds, including the birds in this study (Figure 2) [4,5,7,17,35,36,46,50,51]. The number of epithelial cell layers varies between species, and among the birds studied here, a variation from two to six layers was observed (Table 3). In the Little Penguin (*Eudyptula minor*), five to six layers were found in the epithelium [36]. The African Penguin (*Spheniscus demersus*) was found to have four layers of epithelial cells and an epithelial thickness of 15 µm [35]. Raptor epithelium ranges from two to five layers thick [17], except in the Golden Eagle, where an epithelium of eight layers and 50 µm of thickness was found [42]. In this study, the thickest epithelium was observed in the Greater Rhea (*Rhea americana*) (a strictly terrestrial and diurnal bird), with up to six epithelial layers, and the thinnest was the Stygian Owl and Common Potoo (nocturnal birds), having a maximum of four lines. The Greater Rhea’s cornea also had a high epithelial proportion, with respect to the total thickness of the cornea, at 10.3% (Figure 4). In a study by Popova et al. (2022), the ratio of the epithelium thickness to the total corneal thickness was similarly defined, where the Hyacinth Macaw (*Anodorhynchus hyacinthinus*) demonstrated the highest epithelial proportion in the group of birds at 9.9% of the corneal thickness [1]. In the present study, the Scarlet Macaw (*Ara macao*) demonstrated that 8% of the total thickness of the cornea corresponds to the epithelium (Figure 4). The Smooth-billed Ani (*Crotophaga ani*) had the highest epithelium vs. total cornea ratio at 16% (Figure 4). The Passeriformes Rufous-bellied Thrush (*Turdus rufiventris*) and Great Kiskadee (*Pitangus sulphuratus*) had epithelium that made up 10.7% and 8.9% of the total corneal thickness, similar to that observed in the Java Sparrow (*Lonchura oryzivora*) (8.9%), also a passerine [1]. Past hypotheses for differential epithelial thicknesses relate to the habitat of different species, where those with a greater risk of trauma (diving birds, birds in arid environments, or those living in dense fauna) may have greater epithelial thickness, particularly in proportion to total epithelial thickness [1,2,5,51,52]. The total size of the bird also likely plays a role in total epithelial thickness.

Bowman’s layer (BL), or the anterior limiting lamina, is a continuous meshwork of condensed collagen fibers located in the anterior stroma beneath the epithelium [7,8,53]. This layer is the corneal structure that has the greatest morphological variation between species, and in some animals, it is not present at all [1,3,10,38,53,54]. More developed mammals have a well-defined BL, as observed in humans and other primates, but it is also present in deer and giraffes [1,53,54,55]. This layer is also described in several species of birds, such as chickens, quails, ducks, pelicans, birds of prey, penguins, parrots, and Passeriformes [1,3,10,17,36,38,42,45,46,56,57]. However, there is no consensus on the function of BL in animals. Popova et al. (2022) and Merindano et al. (2002) consider that BL in birds is rudimentary, as it is not as clearly defined as in primates [1,54]. Alternatively, Kafarnik et al. (2007) described the BL of birds as similar to that of primates (acellular and with homogeneous reflectivity) when observed through in vivo confocal microscopy [3]. This is in corroboration with Gonçalves et al. (2016), who suggested that the chicken cornea is an excellent research model for refractive surgeries in humans due to the similarity of corneal structures, with an emphasis on the Bowman’s layer [45].

**Figure 5:**
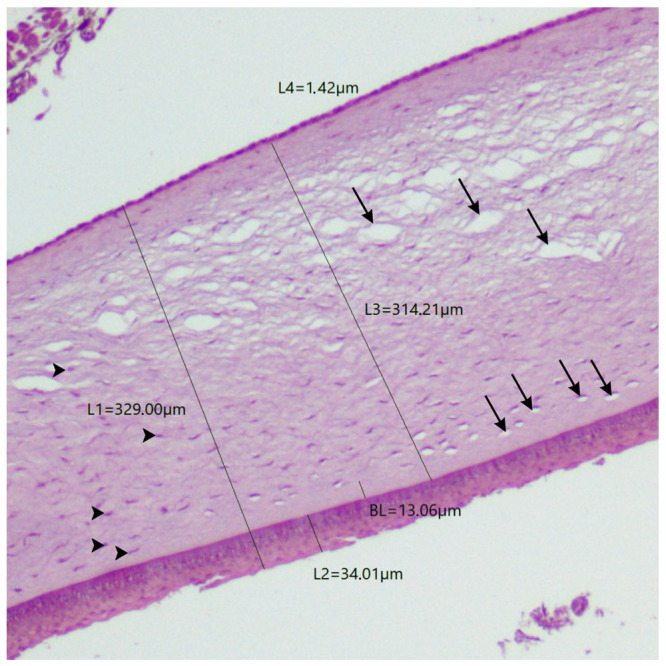
Photomicrographs of the peripheral cornea of the *Pitangus sulphuratus*. Eosinophilic keratocyte nuclei (arrowhead) and artifacts (arrows) are scattered throughout the stroma. Measurements referring to the thickness of the total cornea (L1), epithelium (L2), Bowman’s layer (BL), stroma (L3), and Descemet’s membrane (L4) are shown. Magnification 100×, stained with hematoxylin and eosin.

In this study, the outermost margin of the BL was well-defined at its border to the basement membrane of the epithelium. However, the innermost limit is not as distinct, as the margin is progressively incorporated into the stroma, making it challenging to measure the thickness of this layer. Collin and Collin (2021) describe not having a clear enough definition to measure the extent of the BL in the Little Penguin, but they stated that it is located 5 µm deep in the stroma [36]. In another study, by Sokolenko et al. (2021), the African Penguin’s BL was not described [35]. Among the birds studied here, the Great Kiskadee, the Stygian Owl, the Greater Rhea, and the Rufous-bellied Thrush had a BL with visible anterior and posterior delimitation but with low contrast in relation to the stroma (Figure 1). It was observed in many histological samples of this study that the stroma suffers from the presence of artifacts in its interior caused by the penetration of the processing substances (Figure 5). These artifacts are randomly present in the substantia propria, but they do not extend into the BL. The same pattern was observed in histological images from other studies [1,17,54,58,59]. It is possible to suggest that this happens due to the dense and compact arrangement of collagen fibersin the BL, making it more difficult for them to be sectioned.

The stroma, or substantia propria, is a dense connective tissue meshwork formed by overlapping collagen fibril lamellae aligned parallel to the corneal surface with scattered keratocytes between them. The density, orientation of the lamellae, and the concentration of keratocytes vary between the regions and across the stroma depth, as well as between species [3,7,8,10,51,60,61,62]. In birds, the collagen lamellae are aligned with each other, forming a precise and regular orthogonal arrangement, with a large number of branches promoting anastomosis of the bundles. This arrangement is most evident in the anterior and middle portion of the substantia propria, and it is associated with greater mechanical rigidity and better light transmittance in the UV spectrum—meaning that the sunlight in contact with the cornea is more scattered, decreasing the amount of light entering the eyes [36,45,60,63,64]. Tsukahara et al. (2010) compared the corneas of birds with mammals, demonstrating that birds have a lower density of keratocytes distributed in the stroma, which are more concentrated in the anterior portion [60]. In histological images from this study, it is also possible to identify that the Smooth-billed Ani and the Rufous-bellied Thrush have more keratocytes in the anterior stroma (Figure 5). Birds also have thicker collagen lamellae than mammals, with greater lamellar thickness indicating better light refraction power [60,64]. Another characteristic of the stroma is that it represents the thickest portion of the cornea, measuring up to more than 90% of the total corneal thickness [10,45,51,62,63,64]. In the present study, five birds demonstrated a stromal thickness between 91 and 95% of the total thickness of the cornea, while three birds, the Rufous-bellied Thrush, the Scarlet Macaw, and the Smooth-billed Ani, demonstrated 76, 78, and 79%, respectively (Figure 4).

In most animals, the corneal stroma and total cornea are thinner at the center than at the periphery [18,65,66]. However, some species demonstrate a thinner peripheral cornea than the center, while others do not demonstrate significant differences between the thicknesses of the regions [36,41,49,67,68]. In the present study, the Scarlet Macaw and the Great Kiskadee showed a slightly thinner periphery than the center, with a mean difference of 14 µm. The Rufous-bellied Thrush demonstrated the opposite, with a cornea measuring 13 µm thicker at the periphery. The Stygian Owl, the Toco Toucan, the Greater Rhea, and the Common Potoo have a more pronounced variation between the regions, which is between 128 and 387 µm thicker in the periphery, respectively. Thickness variation between corneal regions and layers, as well as collagen quantity and arrangement, are not well understood [8,64,68,69].

## 5. Conclusions

The histomorphometric description of the corneas of different avian species from Brazil revealed similarities between the corneas of birds and other vertebrates, but with specific differences in the metrics of each species. We would like to emphasize that the analysis of corneal metrics and histomorphology was intended to provide a basic description of the data without aiming to make statistical inferences. Although the data are representative of avian species, they are insufficient for understanding intra- or interspecific variability, as only one individual from each species was studied. Therefore, a better understanding of both the morphology and the function of specific structures can enhance our knowledge of how birds interact visually with their environment and improve our ability to interpret pathological changes in the avian cornea.

## Figures and Tables

**Table 1:** Order, family, and popular names of the bird species studied.

Species	Order	Family	Brazilian Popular Names	Popular Names in IUCN Red List
*Asio stygius* (Wagler, 1832)	Strigiformes	Strigidae	Mocho-preto	Stygian Owl
*Crotophaga ani* (Linnaeus, 1758)	Cuculiformes	Cuculidae	Anu-preto	Smooth-billed Ani
*Pitangus sulphuratus* (Linnaeus, 1766)	Passeriformes	Tyrannidae	Bem-te-vi	Great Kiskadee
*Ramphastos toco* (Statius Muller, 1776)	Piciformes	Ramphastidae	Tucanuçu	Toco Toucan
*Turdus rufiventris* (Vieillot, 1818)	Passeriformes	Turdidae	Sabiá-laranjeira	Rufous-bellied Thrush
*Rhea americana* (Linnaeus, 1758)	Rheiformes	Rheidae	Ema	Greater Rhea
*Ara macao* (Linnaeus, 1758)	Psittaciformes	Psitacidae	Araracanga	Scarlet Macaw
*Nyctibius griseus* (Gmelin, 1789)	Nyctibiiformes	Nyctibiidae	Urutau	Common Potoo

**Table 2:** Measurement of the total corneal thickness and the thickness of its layers in micrometers (µm).

	Cornea Full Thickness		Epithelium		Bowman’s Layer		Stroma		Descemet’s Layer	
Species	Central	Peripheral	Central	Peripheral	Central	Peripheral	Central	Peripheral	Central	Peripheral
*Asio stygius*	254.76	642.2	9.53	8.91	4.3	3.02	236.08	623.19	1.92	2.33
*Crotophaga ani*	172.27	220.75	37.71	26.32	4.24	4.04	131.39	182.40	1.59	1.27
*Pitangus sulphuratus*	341.45	329	25.58	34	10.16	13.06	311.64	314.21	2.01	1.42
*Ramphastos toco*	374.36	721.38	18.65	16.31	6.27	9.3	344.2	694.87	3.06	3.27
*Turdus rufiventris*	179.33	192.25	19	21.09	3	3.34	140.45	143.45	3.14	2.11
*Rhea americana*	327.76	439	39.95	39.03	3.62	4.32	299.71	399.14	4.61	3.17
*Ara macao*	282.88	266.97	21.08	23.43	3.52	7.84	232.06	200.07	19.72	13.94
*Nyctibius griseus*	224.5	352.5	8.5	10	2.81	4.48	206.6	329.44	3.37	1.55

**Table 3:** Number of layers and characteristics of the corneal epithelium from avian species.

	Epithelial Layers	
Species	Central	Peripheral
*Asio stygius*	3 to 4 layers (1 basal, 1 to 2 polyhedral squamous, and 1 flat squamous)	3 to 4 layers (1 basal, 1 to 2 polyhedral squamous, and 1 flat squamous)
*Crotophaga ani*	5 to 6 layers (1 basal, 2 to 3 polyhedral squamous, and 2 flat squamous)	4 to 5 layers (1 basal, 1 to 2 polyhedral squamous, and 2 flat squamous)
*Pitangus sulphuratus*	3 to 4 layers (1 basal, 1 to 2 polyhedral squamous, and 1 flat squamous)	4 to 5 layers (1 basal, 1 to 2 polyhedral squamous, and 2 flat squamous)
*Ramphastos toco*	3 to 5 layers (1 basal, 1 to 3 polyhedral squamous, and 1 flat squamous)	3 to 4 layers (1 basal, 1 to 2 polyhedral squamous, and 1 flat squamous)
*Turdus rufiventris*	3 to 5 layers (1 basal, 1 to 3 polyhedral squamous, and 1 flat squamous)	3 to 5 layers (1 basal, 1 to 3 polyhedral squamous, and 1 flat squamous)
*Rhea americana*	3 to 6 layers (1 basal, 1 to 3 polyhedral squamous, and 1 to 2 flat squamous)	3 to 6 layers (1 basal, 1 to 3 polyhedral squamous, and 1 to 2 flat squamous)
*Ara macao*	3 to 5 layers (1 basal, 1 to 3 polyhedral squamous, and 1 flat squamous)	3 to 5 layers (1 basal, 1 to 3 polyhedral squamous, and 1 flat squamous)
*Nyctibius griseus*	3 to 4 layers (1 basal, 1 to 2 polyhedral squamous, and 1 flat squamous)	3 to 4 layers (1 basal, 1 to 2 polyhedral squamous, and 1 flat squamous)

**Table 4:** Total corneal thickness of previously studied bird species and of the birds studied here, including their sizes/weights, habits and feeding.

Species (Popular Name)	Size and Weight of the Bird ^1^	Habits ^1,2^	Feeding ^1,2^	Total Corneal Thickness	Source
*Eudyptula minor* (Little Penguin)	30 cm 1.1–1.2 kg	Diurnal, amphibious, flightless	Piscivore	380 ± 54 µm (central region)	(Collin & Collin, 2021) [36]
*Spheniscus demersus* (African Penguin)	45 cm 3.1 kg	Diurnal, amphibious, flightless	Piscivore	450 µm (region not specified)	(Sokolenko et al., 2021) [35]
*Spheniscus demersus* (African Penguin)	45 cm 3.1 kg	Diurnal, amphibious, flightless	Piscivore	384 ± 30 µm (central region)	(Gonzalez-Alonso-Alegre et al., 2015) [11]
*Spheniscus humboldti* (Humboldt Penguin)	66–70 cm 4–5 kg	Diurnal, amphibious, flightless	Piscivore	636 µm ^3^ (region not specified)	(Popova et al., 2022) [1]
*Gallus gallus domesticus* (Domestic chickens)	40–60 cm 2580.2 g	Diurnal, terrestrial, domestic	Granivore and insectivore	242 µm (central region)	(Montiani-Ferreira et al., 2004) [16]
*Gallus gallus domesticus* (Domestic chickens)	40–60 cm 2.6–4.5 kg	Diurnal, terrestrial, domestic	Granivore and insectivore	225.3 ± 30 µm (region not specified)	(Gonçalves et al., 2016) [45]
*Coturnix coturnix* (Common Quail)	17.5 cm 70–155 g	Diurnal, terrestrial, grassland	Granivore	154 ± 17.7 µm (region not specified)	(Gonçalves et al., 2016) [45]
*Coturnix japonica* (Japanese Quail)	16–18 cm 90–115 g	Diurnal, terrestrial, grassland	Granivore	138.64 µm (region not specified)	(Mayakkannan et al., 2018) [46]
Ostrich (species not described by the author)	180–270 cm 90–130 kg	Diurnal, terrestrial, flightless	Omnivore	550 ± 35 µm (central region)	(Liu et al., 2016) [12]
*Rhea americana* (Greater Rhea)	1.34–1.70m 26–36 kg	Diurnal, terrestrial, flightless	Omnivore	327.76 µm (central region) 439 µm (peripheral region)	This study
*Harpia harpyja* (Harpy Eagle)	89–102 cm 6–9 kg	Diurnal, raptor, rainforests	Carnivore	563 µm (region not specified)	(Grego et al., 2025) [47]
*Aquila chrysaetos* (Golden Eagle)	70–84 cm 3–6.125 kg	Diurnal, raptor, open or semi-open areas	Carnivore	640 µm (central region) 1200 µm (peripheral region)	(Murphy & Dubielzig, 1993) [42]
*Falcon tinnunculus* (Common Kestrel)	36–58 cm 907 g	Diurnal, raptor, open or semi-open areas	Carnivore	129 µm (central region) Varies from 197 to 210.8 µm (peripheral region)	(Werther et al., 2017) [18]
*Asio stygius* (Stygian Owl)	38–46 cm 400–675 g	Nocturnal, raptor, open or semi-open areas	Carnivore	254.76 µm (central region) 642.2 µm (peripheral region)	This study
*Nyctibius albicollis* (Common Pauraque)	20–30 cm 50–70 g	Crepuscular to nocturnal, open or semi-open areas	Insectivore	146.2 ± 34.5 µm (central region) (149.2 ± 35.8 μm (peripheral region)	(Tozetti et al., 2024) [48]
*Nyctibius griseus* (Common Potoo)	34–38 cm 160–190 g	Nocturnal, open or semi-open areas	Insectivore	224.5 µm (central region) 352.5 µm (peripheral region)	This study
*Columba livia* (Domestic Pigeon)	29–35 cm 315–410 g	Diurnal, domestic, urban areas	Granivore	157 µm (central region) 188 µm and 169 µm (peripheral nasal and temporal regions)	(Chard & Gundlach, 1938) [14]
*Calypte anna* (Anna’s Hummingbird)	10 cm 4–4.5 g	Diurnal, scrub forest	Nectarivore	59 µm (central region) 48 µm (peripheral region)	(Moore et al., 2019) [49]
*Pitangus sulphuratus* (Great Kiskadee)	21–26 cm 52–68 g	Diurnal, rainforests, urban areas	Omnivore	341.45 µm (central region) 329 µm (peripheral region)	This study
*Turdus rufiventris* (Rufous-bellied Thrush)	25 cm 68 g	Diurnal, rainforests, urban areas	Omnivore	179.33 µm (central region) 192.25 µm (peripheral region)	This study
*Lonchura oryzivora* (Java Sparrow)	15–17 cm 24.5 g	Diurnal, open grassland	Granivore	166 ± 5 µm (region not specified)	(Popova et al., 2022) [1]
*Crotophaga ani* (Smooth-billed Ani)	35 cm 115 g	Diurnal, rainforests, urban areas	Omnivore	172.27 µm (central region) 220.75 µm (peripheral region)	This study
*Ramphastos toco* (Toco Toucan)	61 cm 592–760 g	Diurnal, scrub forests	Omnivore	374.36 µm (central region) 721.38 µm (peripheral region)	This study
*Ara macao* (Scarlet Macaw)	89 cm 1.2 kg	Diurnal, rainforests	Frugivore	282.88 µm (central region) 266.97 µm (peripheral region)	This study
*Anodorhynchus hyacinthinus* (Hyacinth Macaw)	1 m 1.2–1.7 kg	Diurnal, rainforests	Frugivore	472 µm ^3^ (region not specified)	(Popova et al., 2022) [1]
*Platalea leucorodia* (Eurasian Spoonbill)	80–90 cm 1.7–2 kg	Diurnal, wetlands	Piscivore	436 µm ^3^ (region not specified)	(Popova et al., 2022) [1]

^1^ https://animaldiversity.org/ (accessed on 20 May 2025); ^2^ https://www.iucnredlist.org/ (accessed on 20 May 2025); ^3^ Approximate mean total corneal thickness.

## Data Availability

The original data presented in the study are openly available in “Corneal Histomorphometry of Birds From the Brazilian Midwest”, Harvard Dataverse, V1, https://doi.org/10.7910/DVN/KGVQVR (accessed on 20 May 2025).
